# Multidrug-Resistant Bacterial Isolates Recovered from Herbal Medicinal Products Sold in Nairobi, Kenya

**DOI:** 10.24248/EAHRJ-D-17-00027

**Published:** 2017-03-01

**Authors:** Richard Korir, Omu Anzala, Walter Jaoko, Christine Bii, Lucia Keter

**Affiliations:** a Kenya Medical Research Institute, Nairobi, Kenya; b University of Nairobi, School of Medicine, Nairobi, Kenya

## Abstract

**Background:**

Medicinal herbs have been reported to be contaminated with microorganisms indigenous to the environment. These microbes become a threat when they harbour drug-resistant traits.

**Objective:**

The aim of this study was to evaluate phenotypic and genotypic drug-resistant traits of bacteria isolated from herbal medicinal products in Nairobi, Kenya.

**Methods:**

We employed an exploratory as well as laboratory-based experimental design. Herbal products were purchased from markets and transported to Kenya Medical Research Institute laboratories for processing and analysis. Microbial contamination and antibiotic susceptibility were determined following standard methods. Antibiotic-resistant genes were determined using polymerase chain reaction. Data were coded and analysed accordingly.

**Results:**

We collected 138 samples of herbal products in the form of liquids, powders, capsules, creams/lotions, and syrups. In total, 117 samples (84.8%) were contaminated with bacteria and 61 (44.2%) were contaminated with fungi. *Bacillus, Klebsiella, Proteus, Staphylococcus, Streptomyces, Escherichia, Enterobacter, Serratia, Yersinia, Morganella, Citrobacter, Erwinia*, and *Shigella* were the bacterial genera identified. Most of the isolated bacteria were generally sensitive to the panel of antibiotics tested, although a few (35 [36.5%]) were resistant; more than half of these were resistant to more than 1 of the antibiotic agents we tested.

**Discussion:**

We found an association between phenotypic and genotypic drug resistance among the drug-resistant bacteria. This study makes it evident that herbal medicinal products sold in Nairobi are contaminated with drug-resistant bacteria.

**Conclusions:**

The results show that herbal medicinal products are a potential source of dissemination of multidrug-resistant bacteria. There is an urgent need for specific education programmes, policies, and regulations that address herbal products' safety to prevent the possibility of these pathogens being involved in deadly invasive infections.

## INTRODUCTION

Antibiotic-resistant bacteria have been a source of an ever increasing therapeutic challenge.^1^ Continued mismanagement of antibiotics and the resulting selective pressure have contributed to the emergence of multidrug-resistant bacteria; this has been regarded as an inevitable genetic response to antimicrobial therapy.^2^ Drug-resistant infectious microbes have become an important public health concern that warrants organisations in public and private sectors worldwide working together.^3,4^ Aside from the public health threat, the search for newer microbial-sensitive treatments to overcome resistant microbes is usually very expensive and contributes to the higher costs of health care, which is attributed to longer hospital stays.^4^

Microbial resistance to antimicrobial agents is usually mediated through gene coding for resistance. The resistant genes are either chromosomal (intrinsic) or plasmid encoded (extrinsic). Plasmids are self-replicating extra chromosomal DNA molecules found in Gram-negative and Gram-positive bacteria as well as in some fungi (yeast and moulds).^5^

Determination of antibiotic-resistant genes through the use of polymerase chain reaction (PCR) techniques provides insights on genetic information relating to resistance to 1 or more antibiotics. The genetic information may also reflect the extent or amount of the multidrug resistance.^6^ Bacteria which are resistant to antibiotic agents may develop anywhere, especially in a confined environment previously contaminated with drug-sensitive bacteria. One such environment can be in herbal medicinal products (HMPs), and HMPs have been previously implicated as a pool for such contamination.^7,8^

The use of HMPs as a form of complementary medicine is becoming increasingly popular in both developing and developed countries.^7^ About 70% to 80% of the world's population, particularly in the developing world, has been shown to depend on herbal drug regimens for their primary health care.^9^ As the pros and cons of HMPs are pondered, it is important to monitor and ascertain their pureness, as HMPs contaminated with microbes, especially drug-resistant microbes, may pose important health, medical, and economic implications.^7^

Monitoring of HMPs will help to identify microbial contamination, provide information on the rate of antimicrobial resistance, and devise mechanisms to slow down the rate of emergence of drug-resistant strains from HMPs.^10^ In the present study, we evaluated selected HMPs from Nairobi, Kenya, for the presence of contaminating microorganisms. These microorganisms were later subjected to susceptibility studies to establish their resistance profiles. The DNA for phenotypic-resistant isolates were extracted and used to determine genotypic resistance using specific primers coding for antibiotic-resistant genes.

## MATERIALS AND METHODS

### Study Site and Design

The study was undertaken in Nairobi, the capital and largest city in Kenya. Nairobi has several herbal clinics, especially in densely populated areas. However, HMPs are also sold in health food stores, pharmacies/chemists, supermarkets, local retailers, and hawkers, among other outlets. This study employed an exploratory as well as laboratory-based experimental design.

### Sample Collection

We collected HMPs from different herbal vendors across Nairobi County. The study sample included 138 different HMPs in different preparations, which included liquids, powders, capsules, creams/lotions, and syrups.

**Figure:**
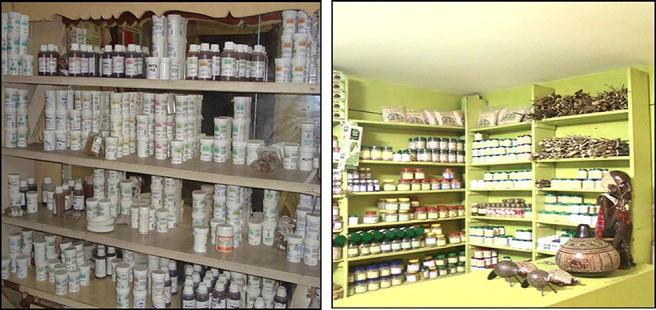
Herbal Products Displayed on Shelves. A, Herbal products sold in herbal chemist or health food stores. B, Herbal products sold in herbal clinic.

**Figure:**
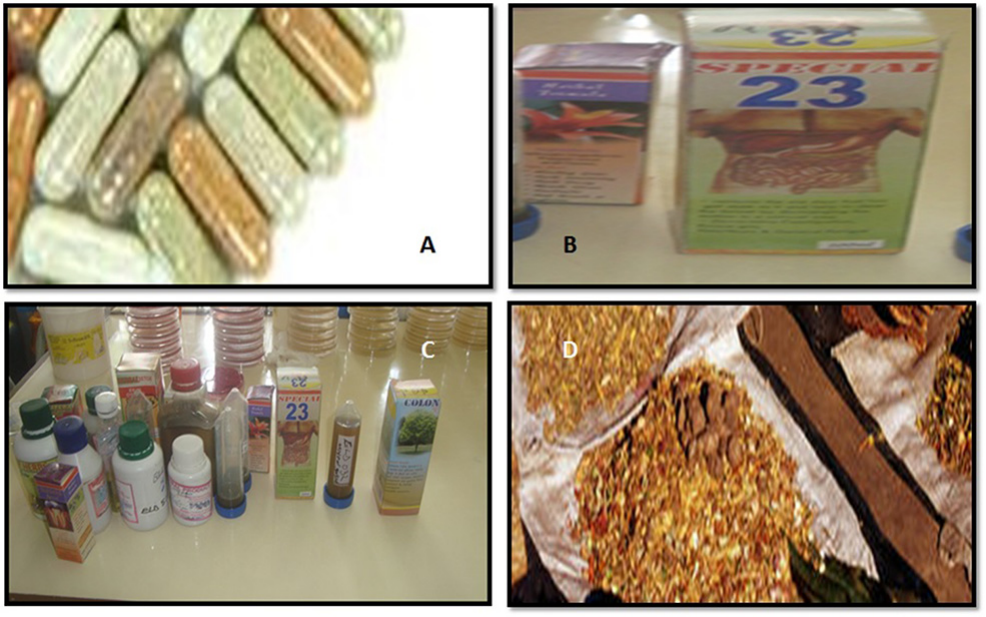
Formulations of Herbal Products. A, Capsules. B, Liquid (concoctions). C, Syrups and concoctions. D, Powdered roots and stem barks.

### Isolation and Identification of Contaminating Bacteria

Each HMP was serially diluted and plated in triplicate on selective, differential, and general purpose media for bacteria growth. HMPs were incubated at 37°C for 12 to 18 hours. The ensuing colonies were further purified, isolated, and characterised using standard methods.^11^

### Susceptibility Testing of the Bacterial Isolates

Briefly, the following antimicrobial discs were placed onto Mueller-Hinton agar plates seeded with the bacteria strains: piperacillin, ciprofloxacin, norfloxacin, cefotaxime, gentamicin, sulphamethoxazole/trimethoprim, chloramphenicol, and ceftazidime. The plates were incubated overnight for 12 to 18 hours, and any microorganism that showed resistance^12^ to any of the antibiotics was isolated for further resistance DNA isolation studies. After isolation, the bacteria were stocked and stored in a negative 40°C deep freezer.^13^

### DNA Extraction, PCR, and Gel Electrophoresis

The bacteria were retrieved from the freezer, thawed, and cultured in brain heart infusion broth at 37°C overnight. Total DNA were extracted from 5 mL of a broth culture grown overnight. After incubation, bacterial cells were harvested by centrifugation at 3,000 rpm (radius 7.20 cm) for 10 minutes; the cell pellets were suspended in phosphate-buffered saline with 100 μg of lysostaphin per millilitre and incubated at 37°C for 30 minutes. The phenol/chloroform extraction method was used for nucleic acid extraction, and the DNA was precipitated in 1 mL of 70% ethanol. The DNA precipitate was dissolved in 50 μl of TE buffer (10 mM Tris-Cl, 1 mM EDTA; pH 8.0) and stored at negative 20°C until processing.^13^

The PCR amplification was performed in a 25 μl reaction mixture (2.5 mL of 10× reaction buffer without MgCl2; 200 μM of each deoxynucleoside triphosphate, 2 mM MgCl2; 2.5 pmol of each primer and approximately 2–4 μl of template DNA) and brought up to a 25 μl final volume with sterile DNA/RNA-free distilled water. To reduce the formation of nonspecific extension products, a ‘hot-start’ protocol was adapted. The PCR reactions were hot-started for 5 minutes at 95°C and placed on ice, and 2 μl of Taq polymerase was added. Reaction mixtures were subjected to 30 PCR cycles (95°C for 2 minutes, then 1 minute at 54°C, and 1 minute at 72°C). A final elongation step of 7 minutes at 72°C was applied in a thermal cycler.^13^

Among the drug-resistant isolates, the following genes were investigated:

- The *aacA-aphD* gene coding for gentamicin resistance, with 227 base pairs. The primers are *aacA-aphD*: F-TAA TCC AAG AGC AAT AAG GGC and *aacA-aphD*: R-GCC ACA CTA TCA TAA CCA CTA.
- The *bla_CMY_* gene, which has 205 base pairs and is responsible forfourth-generation cephalosporin (cefepime, ceftazidime) resistance. Its primers are *bla_CMY_*: F-GAC AGC CTC TTTCTCCAC and *bla_CMY_*: R-TGG AAC GAA GGC TAC GTA.
- The *bla_CTX-M_* single gene coding for cefotaxime and piperacillin resistance, which has 499 base pairs. Its primers are CTX-M1: F3-GAC GAT GTC ACT GGC TGA GC and CTXM1: R2-AGC CGC CGA CGC TAA TAC A.
- The *gyrA* is a single gene that codes for norfloxacin and ciprofloxacin resistance and has 574 base pairs. Its primers are *gyrA*: F1-ATG TCA GAC AAT CAA CAA CAA GC and *gyrA*: R3-ACA TTC TTG CTT CTG TAT AAC GC.
- The *SulA* gene that codes for sulphamethoxazole/trimethoprim resistance and has 360 base pairs. Its primers are *SulA*: F-AC TGC CAC AAG CCG TAA and *SulA*: R-GTC CGC CTC AGC AAT ATC.^14^

The DNA products were loaded on an agarose gel, and gel electrophoresis was performed to separate the mixture of DNA pellets. The DNA bands (products) were visualised using ultraviolet transmission light and were photographed alongside the controls and molecular weight markers.^13^ We then identified the DNA bands in reference to the controls to determine the associated genes. This information was used to correlate the phenotypic and genotypic characteristics of the targeted drug-resistant bacteria.

## RESULTS

We collected and analysed 138 samples of HMPs. These samples included 106 powders (76.8%), 18 liquids (13.0%), 8 syrups (5.8%), 4 creams/lotions (2.9%), and 2 capsules (1.4%). Seventy-four of the samples (53.6%) came from street vendors/hawkers, 34 (24.6%) from herbal clinics, 19 (13.8%) from supermarkets/shops, 7 (5.1%) from manufacturers/wholesalers, and 2 each (1.4%) from chemists and health food stores.

Bacteria isolated from the collected HMPs were grouped into 13 genera: *Bacillus, Klebsiella, Proteus, Staphylococcus, Streptomyces, Escherichia, Enterobacter, Serratia, Yersinia, Morganella, Citrobacter, Erwinia*, and *Shigella*. The genera and species of the bacteria isolated from HMPs are shown in the Table.

**TABLE.** Genus and Specific Epithets of the Isolated Bacteria

Genus	Organism(s)	Gram Reaction	Frequency (%)
*Citrobacter*	*C diversus*	Gram −	3 (100.0)
*Enterobacter*	*E aerogens, E cloacae*	Gram −	22 (100.0)
*Streptomyces*	*S* spp.	Gram +	74 (100.0)
*Bacillus*	*B anthracoides, B* spp.	Gram +	64 (100.0)
*Erwinia*	*E chrysanthemi*	Gram −	1 (100.0)
*Escherichia*	*E coli*	Gram −	7 (100.0)
*Morganella*	*M morganii*	Gram −	2 (100.0)
*Klebsiella*	*K pneumoniae*	Gram −	4 (100.0)
*Proteus*	*P penneri*	Gram −	25 (100.0)
*Serratia*	*S fonticola, S marcescens, S rubidaea*	Gram −	14 (100.0)
*Shigella*	*S sonnei*	Gram −	1 (100.0)
*Staphylococcus*	*S aureus*	Gram +	5 (100.0)
*Yersinia*	*Y enterocolitica*	Gram −	11 (100.0)
*Total*			233 (100.0)

Abbreviations: Gram −, gram negative; Gram +, gram positive.

For this study, we tested 96 (100.0%) isolates of bacteria for susceptibility to the commonly used antibiotics. Most of the isolated bacteria (61 [63.5%]) were generally sensitive to the panel of antibiotics. Thirty-one (32.3%) bacterial isolates were resistant to ceftazidime; 33 (34.4%) were resistant to cefotaxime; 2 (2.1%) were resistant to gentamicin; 5 (5.2%) were resistant to chloramphenicol; 1 (1.0%) was resistant to piperacillin; and 2 (2.1%) each were resistant to norfloxacin and ciprofloxacin, respectively.

The isolated bacteria were resistant to either 1 or more than 1 antibiotic. The following isolates were resistant to only 1 antibiotic: *Morganella morganii* (MM2), resistant to chloramphenicol; *Enterobacter cloacae* (EC3), resistant to ceftazidime; and *Proteus penneri* (PP20), resistant to cefotaxime. Four isolates were resistant to 3 antibiotics: *Citrobacter diversus* (CD1), resistant to gentamicin, cefotaxime, and norfloxacin; *M morganii* (MM1), resistant to sulphamethoxazole/trimethoprim, chloramphenicol, and cefotaxime; *Enterobacter aerogenes* (EA2), resistant to ceftazidime, cefotaxime, and piperacillin; and *Klebsiella pneumoniae* (KP2), resistant to sulphamethoxazole/trimethoprim, ceftazidime, and cefotaxime.

Two isolates exhibited resistance to 4 antibiotics: *C diversus* (CD2), resistant to sulphamethoxazole/trimethoprim, chloramphenicol, norfloxacin, and ciprofloxacin; and *E cloacae* (EC5), resistant to sulphamethoxazole/trimethoprim, ceftazidime, cefotaxime, and norfloxacin. Twenty-six isolates were resistant to 2 antibiotics. Most of these isolates (22 [62.9%]) were resistant to both ceftazidime and cefotaxime.

Bacterial DNA extraction was performed and was later amplified independently with reverse and forward primers in a single reaction in order to determine antibiotic-resistant genes among the phenotypic-resistant isolates. The bacteria were *C diversus* (CD1 and CD2), *E aerogenes* (EA2), *E cloacae* (EC2, 3, 4, 5, 8, 9, and 10), *Erwinia chrysanthemi* (ERC1), *K pneumoniae* (KP2), *M morganii* (MM1 and 2), *P penneri* (PP4, 8, 9, 10, 11, 12, 13, 17, 18, 20, 21, and 22), *Serratia marcescens* (SM1 and 2), *Serratia rubidaea* (SR4, 6, and 7), and *Yersinia enterocolitica* (YE3, 6, 7, and 8).

Except for 2 isolates, all the bacteria found to be resistant to the drugs were found to contain drug-resistant genes. There was an association between the phenotypic and genotypic drug resistance among the drug-resistant isolates. All the isolates that were phenotypic resistant to cefotaxime and ceftazidime contained the *bla_CTX-M_* gene and the *bla_CMY_* gene.

**FIGURE 1.**
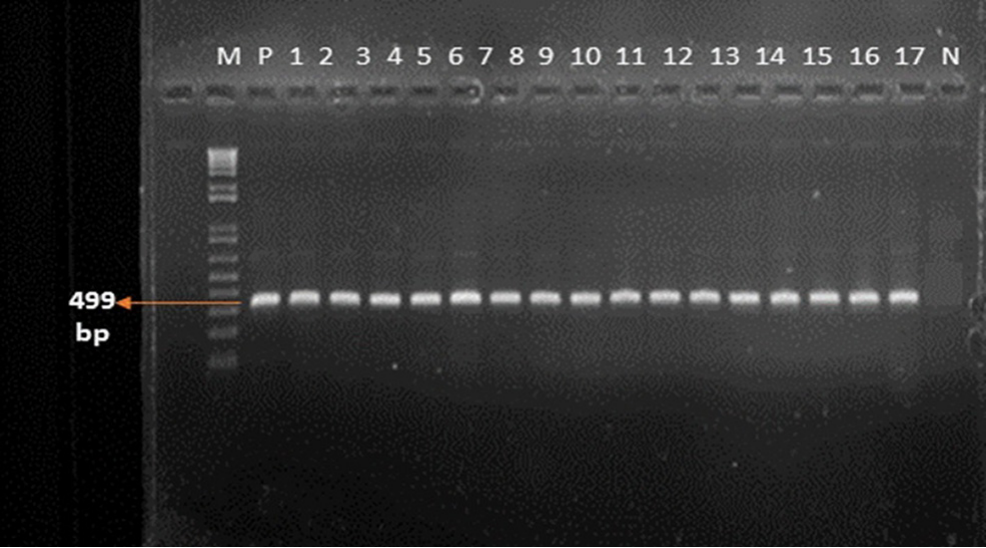
Bacterial Isolates with *bla_CTX-M_* Gene Coding for Cefotaxime Resistance with 499 Base Pairs

**FIGURE 2.**
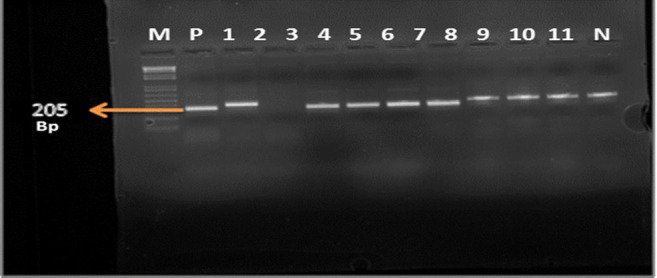
Bacterial Isolates with *bla_CMY_* Gene Coding for Ceftazidime Resistance with 205 Base Pairs

Figure 1 shows DNA fragments for isolates that were resistant to cefotaxime; the *bla_CTX-M_* gene has 499 base pairs. Figure 2 shows DNA fragments for isolates that had genes coding for ceftazidime resistance; the *bla_CMY_* gene has 205 base pairs.

Lane M is a molecular marker with different bands denoting different molecular weights: each band stands for 150 base pairs. Lane P is the positive control; Lane N is the negative control; and Lanes 1–17 show bacterial isolates containing the *bla_CTX-M_* gene. The *bla_CTX-M_* gene has 499 base pairs.

Lane M is a molecular marker with different bands denoting different molecular weights: each band stands for 50 base pairs. Lane P is the positive control; Lane N is the negative control; and Lanes 1–11 show bacterial isolates containing the *bla_CMY_* gene. The *bla_CMY_* gene has 205 base pairs.

## DISCUSSION

HMPs designed for chemotherapeutic and pharmacological benefits should be effective against the targeted medical condition. Several factors could compromise this goal, including contamination with pathogenic and nonpathogenic micro-organisms.^15^ Apart from possible microbial degradation of the active constituents contained in the HMPs, the presence of contaminating microorganisms could constitute a source of infection and a serious health risk to consumers, who were probably already overwhelmed by the serious medical conditions for which the HMPs were initially indicated.^16^ Drug-resistant traits in products that are consumed can lead to serious health conditions which do not respond to antibiotic agents.

In this study, soil bacteria formed the bulk of the isolates found. These bacteria were *Streptomyces* species (74 [53.6%]) and *Bacillus anthracoides* (64 [46.4%]), which indicate environmental contamination. According to a study done by Grierson,^17^ *B anthracoides* is pathogenic to guinea pigs and mice under experimental conditions, and it would appear to occupy a position between the virulent *Bacillus anthracis* and the nonpathogenic members of the group of aerobic sporing bacilli (e.g., *Bacillus subtilis, Bacillus mesentericus*). When people who are sick are exposed to *B anthracoides* through consumption of contaminated HMPs, this could pose a serious health risk.

*Escherichia coli* were isolated in liquid HMP formulations, which include concoctions, decoctions, and infusions. Generally all the liquids were dissolved in water; hence the water for dissolution might have contained *E coli*. *E coli* is an indication of faecal contamination and is associated with gastroenteritis.

The most important bacteria we isolated in terms of potential human diseases were *K pneumoniae, Staphylococcus aureus, Proteus species, Shigella sonnei*, and *E coli* (among other coliforms). These results concur with a similar study by Frazier and Westhoff,^18^ who isolated bacteria of clinical importance such as *Bacillus* species, *Salmonella* species, and *E coli* from herbal products, although the current study did not find any species of *Salmonella*. Shukla and colleagues,^19^ in a similar study, reported a high recovery rate of these suspected infectious bacteria from indigenous orally consumed herbal medications. Danladi and colleagues^20^ found similar results in their study on herbal preparations. However, these other studies did not determine drug susceptibility of the isolated bacteria.

The majority of the bacterial isolates found in this study (61 [63.5%]) were sensitive to the antibiotics tested. These results concur with a study done by Alwakeel on microbial contaminants of herbal medicine, where he found that most (75%) of the bacteria isolated were sensitive to the antibiotics,^21^ but did not determine the presence of drug-resistant genes.

Testing bacterial pathogens for their responses to chemo-therapeutic agents is common practice in clinical and food microbiology.^22^ In our study, 36.5% of the isolated bacteria were resistant to the panel of antibiotics we tested. Other studies have observed a higher level of resistance (46.2% to 51.7%) to the commonly used antibiotics.^22,23^ Angulo and colleagues^24^ alleged that the ability of bacteria to evolve mechanisms to resist attack by antimicrobials was recognised soon after the widespread deployment of the first antibiotics. DeWaal and colleagues^25^ have also suggested that resistance is an inevitable consequence of antibiotic use; the more antibiotics are used, the more bacteria will develop resistance to them.

All the bacteria we found to be drug-resistant, except for 2 (5.7%) isolates, were found to contain resistant genes. The antibiotic-resistant bacterial isolates were resistant to either 1 or more than 1 of the antibiotics tested and were also found to contain drug-resistant genes. There was a direct association between phenotypic resistance and genotypic resistance.

Antibiotics are used to treat bacterial infections. They may be used as a short- or long-term treatment, depending on whether the problem is acute or chronic. A study by Ash and colleagues^26^ found that bacteria with intrinsic resistance to antibiotics are found in nature. Such organisms may acquire additional resistant genes from other bacteria introduced into soil or water, and the resident bacteria may be the reservoir or source of the widespread drug-resistant organisms found in many environments. In bacteria, antimicrobial resistance is facilitated by the ability to quickly adapt to new environments and to replicate very quickly. From this comes the aptitude to mutate the DNA acquired from other drug-resistant bacteria.^4^

The acquisition of resistance to drugs may be due to chromosomal mutations or mobile genetic elements like plasmids that are often capable of transfer from one strain of organism to another, even across the species barrier. Plasmid transfer within and across species is further enhanced through the activities of transposons, which are mobile genetic elements that can confer resistance determinants.^5^ The ability of transposons to integrate into either conjugative plasmids or into an organism's chromosomes enhances the transferability of a given determinant of resistance.^25^

This process is a natural phenomenon exacerbated by the abuse, overuse, and misuse of antimicrobials in the treatment of human illness and in animal husbandry, aquaculture, and agriculture.^2^ When drug-resistant organisms are present in medicaments, such as HMPs, they could behave as opportunist pathogens and initiate an infection, particularly in immune-compromised patients. They can also lead to transfer of antibiotic-resistant traits to hitherto drug-sensitive microorganisms that cohabit within the consumers of the contaminated products.

Given the increasing rate of development of resistant bacteria strains, the main challenge is to slow the rate at which resistance develops and spreads. To do this, physicians, pharmacists, researchers, and consumers alike need to be more aware of the selective pressures that drive these bacteria to decrease their susceptibility.^2^ These selective pressures include the abuse, overuse, and misuse of antimicrobials in therapy; improper manufacturing and mis-handling of HMPs;^2,5^ and numerous other socioeconomic factors that govern the development of multidrug-resistant bacteria strains.^26^ In such circumstances, a collective and concerted effort towards preventing the development of resistant bacteria strains through rational antimicrobial use policy, right practices, and intensive research leading to novel and alternative drug therapies would help check the emergence of multidrug-resistant bacteria strains.

## CONCLUSION AND RECOMMENDATIONS

The results of the present work show that HMPs were contaminated with both pathogenic and nonpathogenic bacteria. Of concern was the multidrug resistance found among the isolated bacteria, since only 3 isolates were resistant to only 1 drug, while 32 isolates were resistant to more than 1 antibiotic. All the drug-resistant bacteria harboured drug-resistant genes. The high rate of strains with multidrug resistance that were isolated from these herbal preparations may indicate widespread antibiotic resistance among microorganisms from different sources. It is therefore important that quality assurance is built into the whole process of manufacturing HMPs. Thus, there is a need for constant monitoring and control of the microbial standards of herbal medicines available on the market. Further studies should sequence bacteria that are found to have genotypic resistance in order to determine their relatedness. Mobile-resistant genes should be determined, because bacteria that share the same environment can transfer mobile genes to antibiotic-sensitive bacteria through plasmids and transposons.
